# Gender inequality in early initiation of breastfeeding in Bangladesh: a trend analysis

**DOI:** 10.1186/s13006-020-00259-y

**Published:** 2020-03-16

**Authors:** Kanchan Kumar Sen, Taslim Sazzad Mallick, Wasimul Bari

**Affiliations:** Department of Statistics, University of Dhaka, Dhaka, 1000 Bangladesh

**Keywords:** Breastfeeding, Early initiation, Gender, Inequality, Trend analysis

## Abstract

**Background:**

Early initiation of breastfeeding within 1 hour after birth is essential for newborns, because it reduces risk of neonatal mortality and hypothermia to a great extent and also helps in preventing the long-term chronic diseases and in increasing energy and immunity to newborn. In order to reach the ‘very good state’ of timely or early initiation of breastfeeding recommended by WHO, Bangladesh needs to increase the current rate of 51.24 to 100%. An attempt has been made in this study to examine how the early breastfeeding practice changes among male and female children with time controlling the factors associated with this practice.

**Methods:**

Data from last four consecutive Bangladesh Demographic and Health Surveys (BDHS) have been used in the study. The participants were included whose child born within the last 5 years preceding the surveys of 2004, 2007 and 2011, and within the last 3 years preceding the survey of 2014 in the study and the respective selected participants were 5145, 4765, 7099 and 4370. To conduct the trend analysis, the descriptive statistics of selected variables along with prevalence of early initiation of breastfeeding have been computed by different years and a multiple logistic regression model has been fitted to the pooled dataset of 2004–2014 considering survey years as time.

**Results:**

Rate of early initiation of breastfeeding increased as time progressed and it was faster for female child compared to male child. For example, female children were significantly 10 and 6% less likely to be initiated early than their counterparts in 2004 and 2007, respectively; whereas after 2007 both male and female children were equally treated for breastfeeding practice. It was also found that rate of early initiation significantly increased for one unit increased in survey year and this increasing rate was higher for female child compared to male child. For example, for one unit of increased in survey year, the early initiation of breastfeeding increased by 60% for male child and by 67% for female child. Besides, survey time, gender, education of parents, wanted index child, mode of delivery, antenatal care visits, wealth index, exposure to media and division were found to have potential influence on early initiation of breastfeeding.

**Conclusion:**

Demographic and health surveys conducted in Bangladesh since 2011 have shown no evidence of gender discrimination regarding timely initiation of breastfeeding. In order to achieve the target rate of early initiation of breastfeeding recommended by WHO, it requires to take effective intervention regarding maternal and child health education.

## Background

For the better health of newborn, it is essential to start initiation of breastfeeding within 1 hour after birth [1, 2]. Early initiation, exclusive and continued breastfeeding through 23 months reduce neonatal and child mortality significantly [3, 4]. An Indian study showed that the women who did not breastfeed their babies within 1 hour after birth had 2.93 times odds of neonatal death compared to women who breastfed within 1 hour after birth [5]. A study conducted in rural Ghana on the impact of early initiation of breastfeeding (EIBF) on newborn deaths showed that initiation of breastfeeding within the first hour of birth could prevent 22% of neonatal deaths [6]. Based on the studies in Ghana, India, and Nepal, a systematic review argued that a 44–45% reduction in the relative risk (RR) of neonatal mortality could be achieved if EIBF within 24 h of birth have been confirmed [7]. Since mother’s milk contains many anti-infective factors and cells, high quality protein, carbohydrate, fats, vitamins and minerals [3], it acts as a protective against infection, diarrhea, pneumonia and birth asphyxia for newborn. It also helps in improving nutritional status, growth and development of infant; in preventing the long-term chronic diseases and in increasing energy and immunity to newborn [8]. EIBF also helps mother to produce breast milk and to reduce postpartum blood loss by releasing oxytocin.

It is recommended by WHO and other child health organizations that an infant should be breastfed within 1 hour after birth and it is considered as the base of healthy development of child as well as the foundation of development of a country [2, 3, 9]. Currently, only 45 and 42% of newborns worldwide and in South Asia, respectively are put to breast within 1 hour after birth [10]. Though an increasing pattern was observed in EIBF in Bangladesh, only about half of mothers (51%) breastfed within 1 hour in 2014; while these rates are 47, 41 and 24% in 2011, 2007 and 2004, respectively [11–14].

The one of the major aim of sustainable development goal 3 (SDG 3) is to reduce the neonatal mortality to below 12 per 1000 live births and under-5 mortality to below 25 per 1000 live births. But Bangladesh is still far away of achieving these targets as these rates are 23 and 38 per 1000 live births, respectively in 2015 [15]. It is well established that neonatal and child mortality can be reduced to some extent by initiating breastfeeding within 1 hour of birth of child. In a study conducted in Bangladesh, it is found that the gap in putting baby to the breast within first hour after birth was 76% [16] and the present gap in Bangladesh is still high, which is 49% [14].

In developing countries, it has been observed that female child was less likely to be breastfed, suggesting an impact of son preference on breastfeeding [17, 18]. This might happen because of socio-economic conditions of the society, religious and cultural beliefs, low literacy rate, male’s dominance as the earning head of the household, old-age support expecting only from son, demand of dowry, etc. [19–22]. Consequence of son preference has also been observed on contraceptive use, abortion, fertility, child mortality [23, 24]. The gender related differences for exclusive breastfeeding, complementary feeding practice and child care have also been addressed in some studies conducted in Bangladesh [25, 26]. This gender discrimination is harmful and includes unethical practices such as female infanticide and prenatal sex selection (selective abortion).

The aim of sustainable development goal 5 (SDG 5) is to eliminate all forms of discrimination against women and girls. One of the objectives of this study is to examine the gender discrimination regarding EIBF in Bangladesh by exploring how this practice changes between male and female newborns across different Bangladesh Demographic and Survey (BDHS) years, 2004–2014.

Several studies conducted in developing countries have identified a number of socio-economic and demographic factors associated with EIBF such as place of residence [27], education of parents [8, 27–34], birth order [8, 27, 35–39], place of delivery [8], mode of delivery [8, 28, 29, 34, 39–58], size of baby [8], use of antenatal care [8, 28, 42, 44, 53, 59–62], working status of mothers [43], wealth index [30, 39, 49, 63, 64], exposure to media [65] and ecological region [8]. A study on rural area of Bangladesh also revealed that the mothers who got assistance from the trained traditional birth attendant or community volunteers during their pregnancy had higher rate of EIBF compared to their counterparts [66].

This study also aims to determine the rates of EIBF by different background characteristics and to identify potential factors influencing EIBF so that decision makers can take proper steps to increase the rate.

## Methodology

### Data and variables

This study used the data from the last four consecutive Bangladesh Demographic and Health Surveys (BDHS) of 2004, 2007, 2011 and 2014, which were nationally representative studies in Bangladesh [11–14]. These surveys used two stage cluster random sampling based on the enumeration areas and households samples. In the first stage, enumeration areas were selected and households were selected from each enumeration areas in the second stage. Details of enumeration areas, sampling design, sampling frame, list of questionnaires were reported in publicly available reports of the mentioned BDHS surveys. The information of the mother and last child born within the last 5 years preceding the surveys of 2004, 2007 and 2011, and within the last 3 years preceding the survey of 2014 were included in the study. The original dataset had information on 11,440, 10,996, 17,842 and 17,863 mothers from the BDHS surveys of 2004, 2007, 2011 and 2014, respectively; we, however, have selected 5145, 4765, 7099 and 4370 mothers from the respective BDHS surveys. Therefore, all together the study represents a total of 21,379 mother-child pairs after combining the data sets of four consecutive BDHS surveys from 2004 to 2014.

Since the objective of this study is to investigate EIBF practices and their determinants, response variable EIBF was defined following WHO guidelines [9]. When a child was put to breast within 1 hour of birth, it was considered that he/she initiated early breastfeeding. In the original data set, responses were recorded in number of hours or days. The response variable has been categorized into two categories: initiation of breastfeeding within the 1 hour of birth (early initiation) and after 1 hour of birth (late initiation). Along with covariates identified in previous studies, some other covariates were also included in the present study.

Age of mother at birth was grouped into three categories: aged below 20, aged between 20 and 30 years, and aged above 30 years. Education of parents was considered as a covariate with three categories: both are uneducated, father/mother is educated and both are educated. Wanted index child was categorized as yes and no. Numbers of antenatal care (ANC) visits have been categorized into no ANC visits and at least one ANC visits during pregnancy. Place of delivery was categorized as at health facility and at home. The wealth index variable, calculated by principal component analysis of available assets during survey, was categorized into three categories: poor (1st and 2nd quintiles), average (3rd quintile), and rich (4th and 5th quintiles).

Before 2010, Bangladesh had six divisions, namely: Barisal, Chittagong, Dhaka, Khulna, Rajshahi and Sylhet. In 2010, Rajshahi division was divided into two divisions as Rajshahi and Rangpur, and hence Bangladesh currently has seven divisions. Since Rangpur division was a part of Rajshahi division, in the data extracted from BDHS 2011 and 2014, Rangpur was merged into Rajshahi division for computational simplicity. This covariate was considered to examine the regional influence on the response variable. Area of residence of mothers has two categories: rural and urban. The gender of newborn (male, female), birth order of index child (first, second or third, fourth or more), place of delivery (at home, at health facility), and mode of delivery (caesarean, normal) were considered in this study. The exposure to media was assessed based on three indicators: listening radio, watching television and reading newspaper and magazines. It was categorized into two categories: whether the respondent was exposed to any of the above media or not.

### Statistical analysis

In this study, data analysis was carried out using STATA statistical package (version 14). The distribution of EIBF by the different BDHS surveys and by different background characteristics of mother and child were reported as percentages. For bivariate and multiple regression analysis, the pooled data set of four consecutive BDHS surveys from 2004 to 2014 has been used. Bivariate analysis was conducted by cross tabulating EIBF by each of the considered covariates to examine the association with Chi-square test. To find the adjusted effects of covariates, binary logistic regression model was used as the dependent variable, EIBF, was binary. Covariates found to have significant association at the bivariate analysis were taken into the regression model. Note that based on previous studies and importance, a few covariates were included in the regression analysis, though they had no significant association at bivariate analysis. Adjusted odds ratios (AOR) with their 95% confidence interval (CIs) were reported in the study along with *p*-values.

## Results

### Univariate analysis

There were a total of 21,379 mother-child pairs from the pooled data of four consecutive demographic and health surveys. Table 1 shows the percentage distributions of some background characteristics of women for previous four consecutive BDHS surveys as well as for the combined sample. The percentages of EIBF within 1 hour of birth were 25.07, 42.67, 47.02 and 51.24% in 2004, 2007, 2011 and 2014, respectively. EIBF thus increased over the time in Bangladesh. About 42% of mothers initiated breastfeeding to their newborn babies within 1 h of birth in the combined sample. Most of the women were in age group 20–30 years and the ratio of women from rural to urban were close among different BDHS surveys, 2004–2014. In particular, at any survey, approximately one-third of the women were from urban area. It was observed that literacy rate of parents increased as time passed, with those with no education decreasing from about half to about 1/3. The distribution of birth order revealed the fact that a greater control in reducing family size in recent years was achieved. It was found that percentage of mothers with four or more births reduced from 27.93% (2004) to 13.75% (2014) i.e., almost 50% reduction was observed in higher birth order. The sex ratio of newborns remained close to 50% over the period of time. More than three-fourth of overall mothers (86.34%) wanted the index child indicating that the missed opportunity of contraceptive use has decreased. Though the percentage of mothers delivering in health facilities increased, still more than 50% of mothers deliver at home. The percentage of mothers who gave birth by caesarean section rapidly increased from 4.94% in 2004 to 24.31% in 2014. A positive trend was observed in antenatal care seeking behavior of mothers in Bangladesh. More than 60% of women had exposure to at least one of the media.

**Table 1:** Distributions for several background characteristics of mothers and children

Background Characteristics	BDHS Surveys				
	2004 (*N = 5145)* %	2007 *(N = 4765)* %	2011 *(N = 7099)* %	2014 *(N = 4370)* %	Pooled *(N = 21,379)* %
**Initiation of Breastfeeding**					
Early initiation (within 1 h)	25.07	42.67	47.02	51.24	41.63
Late initiation	74.93	57.33	52.98	48.76	58.37
**Maternal Age at Birth**					
**<** 20	27.11	25.69	25.82	28.05	26.56
20–30	57.01	57.92	60.54	59.63	58.92
30+	15.88	16.39	13.64	12.31	14.52
**Gender of Newborn**					
Male	50.86	51.04	51.70	51.60	51.33
Female	49.14	48.96	48.30	48.40	48.67
**Area of Residence**					
Urban	31.39	35.66	31.40	32.31	32.60
Rural	68.61	64.34	68.60	67.69	67.40
**Parent’s Education**					
Both uneducated	53.13	46.28	38.79	33.08	42.74
Father/Mother educated	22.21	23.42	25.92	26.99	24.69
Both educated	24.66	30.30	35.29	39.93	32.57
**Birth Order**					
First	28.24	31.29	34.02	40.73	33.39
Second/Third	43.83	44.22	47.36	45.51	45.33
Forth/Higher	27.93	24.49	18.62	13.75	21.17
**Wanted Index Child**					
No	15.26	15.42	13.49	10.12	13.66
Yes	84.74	84.58	86.51	89.88	86.34
**Place of Delivery**					
Health Facility	34.79	41.28	28.71	40.07	35.30
Home	65.21	58.72	71.29	59.93	64.70
**Mode of Delivery**					
Normal	95.06	90.26	84.04	75.69	86.37
Caesarean	4.94	9.74	15.96	24.31	13.63
**ANC Visits**					
No	41.75	37.52	33.20	21.14	33.76
Yes	58.25	62.48	66.80	78.86	66.24
**Wealth Index**					
Poor	40.33	39.06	39.99	39.68	39.80
Average	18.39	18.45	19.19	19.22	18.84
Rich	41.28	42.50	40.82	40.82	41.36
**Exposure of Media**					
No	30.15	36.22	34.39	37.58	34.43
Yes	69.85	63.78	65.61	62.42	65.67
**Division**					
Barisal	11.64	13.39	11.55	12.01	12.08
Chittagong	20.74	19.94	19.13	19.27	19.72
Dhaka	21.94	21.41	16.81	17.71	19.25
Khulna	13.82	12.76	12.11	11.78	12.60
Rajshahi	20.19	16.96	26.09	24.49	22.31
Sylhet	11.66	15.55	14.31	14.74	14.04

### Bivariate analysis

To examine the unadjusted association between early initiation of breastfeeding and some background characteristics of mothers, a bivariate analysis was conducted using the pooled data obtained from four consecutive BDHS surveys, 2004–2014. The results obtained from this analysis are reported in Table 2. Survey year, age of mother at birth, area of residence, parent’s education, birth order, wanted index child, place of delivery, mode of delivery, wealth index and division were found to have significant association with EIBF. The rate of EIBF within 1 hour of birth became double during a 10-year period. The result also showed that the prevalence of EIBF within 1 hour of birth was higher among mothers who were below 20 years old (42.97%) compared to mothers who were 20–30 (41.71%) or 30+ (38.85%) years old. Educated parents [either father/mother (44.48%) or both (41.82%)] had the higher prevalence of EIBF compared to uneducated parents (39.85%). In terms of birth order, the result showed that the rate of EIBF was significantly higher when child was the first birth (41.46%) or the second/third birth (43.26%) than a child with birth order four or more (38.39%). Mothers who wanted index child had higher prevalence of initiating breastfeeding within 1 hour after birth than the mothers who did not want index child. Surprisingly, it was found that prevalence of EIBF was lower for children born at the health facilities (37.26%) than those born at home (44.01%). EIBF was higher in children born normally than with caesarean section (43.81% versus 27.74%). The highest prevalence of EIBF was in Sylhet division (50.35%) and the lowest in Chittagong (36.54%).

**Table 2:** The prevalence of EIBF for both male and female newborns over the survey years

Percentage of EIBF										
Background Characteristics	Male				Female				Overall	*p*-value
	2004	2007	2011	2014	2004	2007	2011	2014		
**Survey Time**										
2004									25.07	
2007									42.67	< 0.001
2011									47.02	
2014									51.24	
**Gender of Newborn**										
Male	25.53	43.63	47.74	49.58					41.97	0.297
Female					24.60	41.66	46.25	52.67	41.27	
**Maternal Age at Birth**										
**<** 20	25.96	46.98	49.35	48.50	26.09	40.92	48.38	56.68	42.97	
20–30	25.45	41.90	47.25	50.56	24.79	43.49	46.63	50.83	41.71	0.001
30+	25.06	44.50	46.91	49.83	21.39	36.09	40.47	52.57	38.85	
**Area of Residence**										
Urban	25.62	40.48	46.17	45.03	23.91	42.91	43.21	49.42	39.85	< 0.001
Rural	25.49	45.50	48.48	52.19	24.93	41.02	47.63	54.24	42.49	
**Parent’s Education**										
Both uneducated	22.19	43.99	48.44	51.28	21.49	42.11	47.42	56.00	39.85	
Father/Mother educated	27.31	44.83	49.49	54.75	24.91	41.91	49.42	56.37	44.48	< 0.001
Both educated	31.40	42.22	45.64	45.65	30.91	40.75	42.48	47.21	41.82	
**Birth Order**										
First	26.11	41.41	45.40	47.94	27.18	39.14	45.71	50.06	41.46	
Second/Third	26.69	44.50	50.41	51.24	25.71	43.12	47.62	53.76	43.26	< 0.001
Forth/Higher	23.05	44.91	45.78	50.96	20.38	42.19	43.89	57.09	38.39	
**Wanted Index Child**										
Yes	26.90	43.46	47.55	49.53	25.64	42.43	46.66	52.80	42.14	< 0.001
No	17.84	44.56	49.04	52.97	18.86	37.43	43.79	51.57	38.39	
**Place of Delivery**										
Health Facility	29.80	40.97	39.66	39.19	28.15	39.27	38.29	41.86	37.26	< 0.001
Home	23.22	45.60	51.06	57.46	22.73	43.26	49.39	59.48	44.01	
**Mode of Delivery**										
Normal	25.62	45.25	51.54	56.28	24.65	43.59	50.03	59.71	43.81	< 0.001
Caesarean	22.95	29.64	28.76	31.13	22.74	22.27	25.14	29.16	27.74	
**ANC Visits**										
No	21.11	44.53	47.36	54.17	20.77	40.16	48.03	54.50	39.27	< 0.001
Yes	28.79	43.08	47.92	49.73	27.26	42.65	45.36	52.18	42.83	
**Wealth Index**										
Poor	21.99	44.50	48.02	53.79	18.62	42.97	47.79	55.62	41.66	
Average	24.13	45.51	52.97	50.71	25.38	41.89	47.71	55.74	43.51	0.013
Rich	29.88	42.08	45.00	45.78	29.73	40.31	44.06	48.31	40.74	
**Exposure of Media**										
No	23.35	45.53	45.57	53.62	18.34	38.67	47.14	54.33	41.70	0.898
Yes	26.51	42.54	48.86	47.76	27.25	43.36	45.78	51.75	41.61	
**Division**										
Barisal	21.38	47.06	48.85	52.55	23.39	43.55	48.96	52.99	42.60	
Chittagong	22.66	34.73	46.28	41.32	19.38	33.05	42.79	51.20	36.54	
Dhaka	21.89	41.77	43.98	50.12	20.81	43.63	39.65	46.28	37.66	< 0.001
Khulna	24.23	45.26	42.66	41.76	24.43	44.53	39.91	45.67	37.75	
Rajshahi	29.00	45.82	50.36	53.61	30.33	39.95	51.08	56.40	45.73	
Sylhet	37.24	50.75	52.47	58.66	32.45	51.32	53.46	61.30	50.35	

Table 2 presents the prevalence of EIBF for both male and female child at each survey years by selected socio-economic and demographic characteristics. It was observed that, in general, the rate of EIBF increased as survey year progressed irrespective of gender of child.

### Logistic regression analysis

All covariates considered in the bivariate analysis were entered into a multiple logistic regression model to determine the adjusted effects on EIBF. The odds ratio (OR) with 95% confidence interval (CI) and *p*-value are reported in Table 3. To examine how this practice changed over time within male and female children, an interaction term between survey time and gender of child was included in the model. Except age of mother at birth, area of residence and place of delivery, all effects were found to have significant association with the EIBF.

**Table 3:** Adjusted odds ratio (OR) of early initiation of breastfeeding for different socio-economic and demographic factors obtained from logistic regression model

Factors	OR	95% CI for OR
**Survey Time**	1.48***	1.43, 1.54
**Maternal Age at Birth**		
**<** 20	1.00	
20–30	0.94	0.87, 1.02
30+	0.94	0.83, 1.06
**Gender of Newborn**		
Male	1.00	
Female	0.86*	0.74, 0.99
**Area of Residence**		
Rural	1.00	
Urban	0.95	0.89, 1.01
**Parent’s Education**		
Both uneducated	1.00	
Father/Mother educated	1.11**	1.03, 1.19
Both educated	1.12***	1.03, 1.22
**Birth Order**		
First	1.00	
Second/Third	1.13**	1.05, 1.22
Forth/Higher	1.05	0.93, 1.18
**Wanted Index Child**		
No	1.00	
Yes	1.10*	1.01, 1.21
**Place of Delivery**		
Health Facility	1.00	
Home	0.95	0.88, 1.02
**Mode of Delivery**		
Normal	1.00	
Caesarean	0.32***	0.29–0.36
**ANC Visits**		
No	1.00	
Yes	1.14***	1.06, 1.21
**Wealth Index**		
Poor	1.00	
Average	1.07^+^	0.99, 1.16
Rich	1.09^+^	1.01, 1.18
**Exposure of Media**		
No	1.00	
Yes	1.12**	1.05, 1.20
**Division**		
Dhaka	1.00	
Barisal	1.15**	1.03, 1.28
Chittagong	0.86**	0.78, 0.94
Khulna	0.96	0.86, 1.06
Rajshahi	1.23***	1.13, 1.35
Sylhet	1.58***	1.43, 1.75
**Survey Time*×*Gender of Newborn**	1.05	0.99, 1.10

^+^*p* < 0.10; *p < 0.05; **p < 0.01; ***p < 0.001

**Fig. 1:**
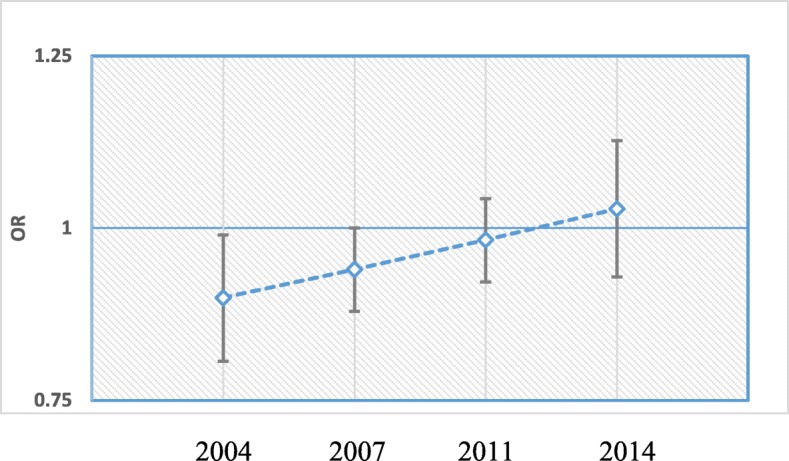
The ORs of early initiation for female vs. male newborn by survey year

**Fig. 2:**
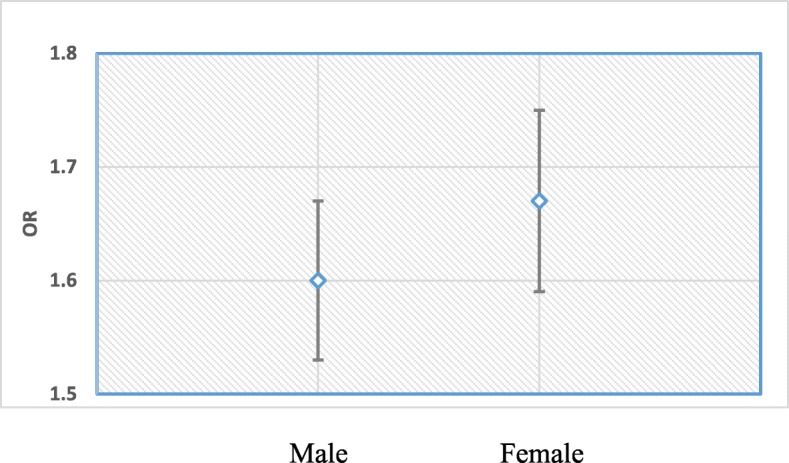
The ORs of early initiation for survey time by gender of newborn

For simplicity in interpretation, survey time was considered as quantitative variable and the values were assigned to the variable as 1 for 2004, 2 for 2007, 3 for 2011 and 4 for 2014. The OR corresponding to main effect survey time was 1.48 [95% CI: (1.43, 1.54); *p*-value< 0.001] and so was 0.86 [95% CI: (0.74, 0.99), *p*-value< 0.05] for the gender of child. The OR of interaction of these two covariates was 1.05 [95% CI: (1.00, 1.10), *p*-value< 0.10]. Figures 1 and 2 were plotted using the adjusted odds ratio and 95% confidence intervals for several survey years and gender of newborn. Odds ratio and standard errors to calculate 95% confidence intervals were computed by using delta method [67]. It was clear from Fig. 1 that OR of EIBF for female child compared to male child increases as time increases. For example, female children were significantly 10% in 2004 and 6% in 2007 less likely to be initiated early than their counterparts; whereas after that both male and female children were treated equally. This implies that gender inequality diminished as time progressed. Figure 2 shows that OR of early initiation significantly increased for each unit increase in time and this increasing rate was higher for female child compared to male child. For one unit of increased in time, the EIBF increased by 60% for male child and by 67% for female child.

If either mother or father were educated, their children was 11% more likely (*p*-value< 0.01) to be breastfed within 1 hour after birth compared to children whose parents were uneducated; this was 12% higher (*p*-value< 0.001) when both mother and father were educated. Children born by caesarean section were 68% less likely to be put to the breast within 1 hour after birth compared to children born normally (*p*-value< 0.001). On the other hand, a significant positive association was observed between early initiation and wealth status; as wealth increased the odds of early initiation increased (*p*-value < 0.10). In addition, women who were exposed to media had 12% higher odds of early initiation as compared to women who were not exposed in any type of media. The women from Barisal, Rajshahi and Sylhet has significantly higher odds of early initiation and women from Chittagong had lower odds as compared to those from Dhaka division.

## Discussion

This study examined the changing pattern of EIBF among male and female newborns over the years of 2004 to 2014 in Bangladesh and assessed the factors associated with EIBF. It showed that the unadjusted rate of early initiation increases with time and by 2014 was 51.24% compared to 25.07% in 2004, an average increase of 10.44% per year. The WHO recommends that all newborns should be breastfed within 1 hour of birth and they referred to EIBF rates of 0–29%, 30–49%, 50–89% and 90–100% to as poor, fair, good and very good, respectively [68]. Findings of this study reveal that Bangladesh made a transition to ‘good’ state in 2014 from ‘poor’ state in 2004, but still almost a 100% increase in EIBF is required in order to achieve the WHO recommendation.

This study revealed the fact that son preference prevailed regarding the EIBF before 2011 in Bangladesh. This discrimination gradually decreased due to a more rapid increase in EIBF rate among female child, this gender inequality in early initiation has been eliminated. This may have happen because of the substantial increase of child health awareness programs in Bangladesh over the last decade. Therefore, it is essential to identify the socio-economic and demographic factors influencing the increase of early initiation to meet the WHO recommendation. For this purpose, a multiple logistic regression was fitted to the pooled data obtained from four consecutive nationally representative surveys.

Except age of mother at birth, area of residence and place of delivery, all other socio-economic and demographic covariates considered into the regression model, were found to be significantly associated with EIBF. The present study demonstrated that the newborn babies of uneducated parents were less likely to be breastfed within the first hour after birth. Note that several earlier studies [8, 27–34] have also found a similar effect of education; however, only mothers’ level of education was considered in those studies, whereas education of both parents was considered in this study. The effect of education may be explained as educated parents have the ability to receive and understand health promotional information easily, and also they can manage the skilled or professional birth assistance or may take decision to go to health facilities during delivery [8]. Another reason might be that mothers or fathers or both who are formally educated may get essential information regarding proper breastfeeding practices from newspaper, magazine or educational institution [27].

Birth order of the newborn child also has a significant effect on early breastfeeding. The babies with the second or third order of births were more likely to be breastfed within 1 hour of birth than the first child, but the babies with the order of four or higher birth did not show the significant evidence to be breastfed early compared to the babies with first order of birth. The possible explanation of this finding is that parents are inexperienced about child caring at the time of having their first baby, on the other hand, for higher order children, parents may face scarcity of resources in terms of time, money, and energy [35]. Similar finding was found in the previous studies of Addis Ababa of Ethiopia [36], Nepal [8], Saudi Arabia [37] and Nigeria [38, 39]. Again, another study in Amibara district of Northeastern Ethopia revealed that mothers with two or three children were less likely to initiate early breastfeeding than mothers with four or more children [27].

Wanted index child was another important factor of early breastfeeding. Babies born by willing parents were more likely to initiate breastfeeding early. This may happen because parents are more willing to follow instructions provided by the health professionals for the better of the newborn children.

Significant association of mode of delivery with EIBF was found in this study. The finding revealed that caesarean babies were less likely to be breastfed within 1 hour of birth compared to the normal babies. There may have several possible causes to delay early breastfeeding in caesarean delivery such as effects/complications of anesthesia, maternal tiredness, respiratory distress among babies, the time-lapse between delivery and the repair of surgical incisions, engaged in lifesaving activities to the mother as well as newborn by healthcare professionals causes late initiation of breastfeeding, post caesarean pain etc. [28, 44, 49, 52, 69]. The negative impact of caesarean delivery on early breastfeeding has also been observed in the several studies [8, 28, 29, 34, 39–58].

Mothers taking antenatal care had higher rates of early breastfeeding. This might be because mothers know the importance of early breastfeeding through the counselling sessions provided by healthcare providers during antenatal care visits [28, 53, 65]. The finding was consistent with other studies [8, 28, 42, 44, 53, 59–62] conducted in several countries.

The study revealed that the babies from rich families were more likely to be breastfed within 1 hour after birth compared to babies from poor families. This may happen due to the fact that rich women may be educated or may access to mass media so that they can understand the benefits of breastfeeding practices. Again, poor women may not have sufficient money to go to health facilities or health centers for taking health care or antenatal care during pregnancy. The studies conducted in Nigeria [39, 49], Indonesia [63], India [30] and Jordan [64] have found similar significant association of wealth index with early breastfeeding.

Mothers who had access to mass media like radio, television, newspaper or magazine were more likely to initiate early breastfeeding than their counterparts. This is because health promotion messages regarding breastfeeding may help mothers to improve their breastfeeding knowledge and practices. A similar finding was seen in Ethiopia [65].

Division, administrative regions of Bangladesh, showed the significant association with early breastfeeding. This could be due to regional differences in breastfeeding practices or in access to health information and health facilities and in what has been said in local media.

Since the study was retrospective, the results obtained in this study may be influenced by recall bias. In some studies, traditional belief was found to have significant association with the EIBF; which has not been included in this study as this variable is not available in BDHS data. Results of this paper do not demonstrate a cause-effect relationship since data used were cross-sectional.

## Conclusion

An increasing trend of EIBF was found in Bangladesh. At present, despite the known benefits of EIBF within 1 hour of birth, only about 51% of mothers initiate breastfeeding within the mentioned time. The factors associated with EIBF were the survey time, gender, education of parents, wanted index child, mode of delivery, antenatal care visits, wealth index, exposure to media and division. An important finding is that the gender inequality in EIBF has diminished and equal preference is currently given for both male and female newborns in families to initiate breastfeeding early. This indicates that Bangladesh has already achieved the SDG 5 goal regarding EIBF. Therefore, it is recommended that the socio-economic and demographic factors influencing the EIBF should be taken into account in any intervention program in order to increase EIBF practices among mothers so that Bangladesh can move from “good state” to “very good state” in terms of EIBF.

## Data Availability

The analysis was based on the datasets collected from Bangladesh Demographic and Health Survey. Information on the data and content can be accessed at. https://dhsprogram.com/data/available-datasets.cfm
